# Use of integrative and complementary practices in Brazil during the COVID-19 pandemic

**DOI:** 10.1371/journal.pone.0311832

**Published:** 2024-12-13

**Authors:** Patricia de Moraes Mello Boccolini, Karine de Lima Sírio Boclin, Islândia Maria Carvalho de Sousa, Cristiano Siqueira Boccolini

**Affiliations:** 1 Faculdade de Medicina de Petrópolis (NIPPIS/FMP/UNIFASE), Núcleo de Informação, Políticas Públicas e Inclusão, Petrópolis, Rio de Janeiro, Brasil; 2 Universidade Estácio de Sá, Rio de Janeiro, Rio de Janeiro, Brasil; 3 Fundação Oswaldo Cruz, Instituto de Pesquisas Aggeu Magalhães, Pernambuco, Brasil; 4 Fundação Oswaldo Cruz, ICICT, LIS, Rio de Janeiro, Rio de Janeiro, Brasil; Health Researcher, BRAZIL

## Abstract

The SARS-CoV-2 coronavirus pandemic posed an unprecedented challenge to global health. In the context of an overwhelmed healthcare system and the rising demand for alternative strategies to manage stress and anxiety, this study aims to investigate and analyze the use of Integrative and Complementary Practices (ICP) in Brazil during the COVID-19 pandemic, emphasizing their importance in promoting health and well-being. We conducted a cross-sectional study among n = 12,136 Brazilian adults predominantly female, white, under 40 years of age and with a predominantly higher education level between August 24 and December 16, 2020, to assess the use of ICP. An online questionnaire was applied, with questions validated in previous health surveys. The sampling method employed was ’virtual snowball,’ post-stratification procedures were used to consider the Brazilian regions, gender, age group, and educational level. The reported prevalence of ICP use was 61.8%, with meditation (28.2%), Reiki (21.7%), herbal medicine (28.2%), and aromatherapy (16.5%) being the most utilized practices. ICPs were more frequently adopted by females, older age groups, individuals with health insurance, and those who practiced social distancing. Health promotion and well-being were the primary reasons for engaging in ICP. The high adherence to ICP during the COVID-19 pandemic in Brazil reflects the population’s search for therapeutic alternatives focused on the well-being and mental health. The utilization of ICP indicates the need to integrate these practices into healthcare systems, considering their potential to complement conventional treatment, especially in times of crisis. Public health policies should recognize and facilitate access to such practices to reduce inequities and promote integrative health. This study contributes to the understanding of the role of ICP in a public health crisis, encouraging further investigation into the potential inclusion of these practices in the healthcare system.

## Introduction

The pandemic caused by the SARS-CoV-2 coronavirus posed an unprecedented challenge to global health. Besides the direct implications of the disease, such as high transmissibility and severity of cases, the psychological and emotional impacts quickly became evident. This scenario led to a growing search for alternative care and well-being strategies to alleviate the psychosocial effects associated with the pandemic [1–3].

Globally, Traditional, Complementary and Integrative Medicine (TCI)) use has significantly increased during the COVID-19 pandemic [1,2,4]. Countries worldwide have reported a surge in adopting various TCI modalities, including traditional Chinese medicine, Ayurveda, herbal medicine, and mind-body practices like Yoga and meditation [4–6]. These practices have been employed to support mental health, boost immunity, and manage stress and anxiety associated with the pandemic [2,4]. The World Health Organization has recognized the potential of these practices in enhancing overall health and well-being, advocating for their integration into public health strategies during crises [7,8]. This global trend underscores the importance of TCI in providing holistic care and addressing the multifaceted challenges posed by the pandemic.

In Brazil the term integrative and complementary practices (ICP) is officialy employed instead of the WHO TCI term [9–11]. Brazil has a National Policy on Integrative and Complementary Practices (PNPIC) within its Unified Health System (SUS) since 2006. This policy supports the integration of practices such as acupuncture, homeopathy, and herbal medicine into public healthcare [10,11]. These practices include a diverse range of approaches, such as herbal medicines, acupuncture, meditation, Yoga, and auriculotherapy, which have proven effective in managing symptoms like anxiety, depression, and stress, often exacerbated during the pandemic [12–14].

There is a significant gap in knowledge about the impacts of ICP during public health crises like the pandemic and how they can influence the population’s well-being and health. Their use can have positive and negative effects, depending on the context and implementation In Hong Kong, a survey found that practices such as the use of vitamins, herbal medicine, and mind-body techniques positively impacted mental and physical health, reducing stress and anxiety, and improving immunity during the pandemic [15]. On the other hand, in Jordan, a study highlighted the risks associated with the use of ICP during the pandemic, particularly among healthcare professionals and students [16]. It was found that a significant number of participants experienced adverse effects probably from these practices [16]. Despite these limitations, the use of ICP is widespread in Brazil [17]. Understanding the patterns of ICP use, including the variety of practices and their application during the COVID-19 pandemic in Brazil, provides a knowledge base that can guide future interventions and organize health services in similar public health crises.

This study aims to investigate and analyze the patterns of ICP use during the COVID-19 pandemic in Brazil, focusing on understanding their importance as tools for health care and the well-being of the Brazilian population, especially considering the strain on the health system and the increased demand for alternative strategies to manage stress, anxiety, and other health issues arising from the pandemic.

## Materials and methods

### Study design and participants

This epidemiological survey evaluated the use of Integrative and Complementary Practices (ICP) among Brazilian adults during the early months of the COVID-19 pandemic. The research was coordinated by the Oswaldo Cruz Foundation (Fiocruz) in partnership with researchers from the Faculty of Medicine of Petrópolis/Arthur Sá Earp Neto University (FMP/UNIFASE) and the Observatory of Integrative and Complementary Practices in Health (ObservaPICS). Data collection extended from August 24 to December 16, 2020, encompassing individuals aged 18 and older residing in Brazil.

### Study instrument

An electronic questionnaire was developed using the RedCap (Research Electronic Data Capture) platform for data collection. The survey instrument consisted of questions adapted from recognized national surveys, including the National Health Survey [18] for sociodemographic characteristics and chronic diseases and the ConVid behavioral research survey [19] for behaviors during the COVID-19 epidemic and infection. Specific questions about the utilization of ICP were adapted from the International Complementary and Alternative Medicine Questionnaire (I-CAM-Q) [20]. The questionnaire was structured in blocks to address sociodemographic characteristics, health conditions, access to health services during the social isolation period, health conditions, and symptoms suggestive of COVID-19 infection. The questionnaire comprised 46 questions in Brazilian Portuguese and is available at https://www.synapse.org/Synapse:syn25148326. The primary outcome investigated was the use of integrative and complementary practices officially recognized by the Ministry of Health, including modalities such as yoga, Reiki, homeopathy, and herbal medicine, among others [11].

### Study sample size

The participant invitation strategy was based on chain sampling [21], starting with the selection of n = 40 researchers from different states in Brazil, who, in turn, invited individuals from their social networks, following stratification by sex, age group, and educational level, and initially totaling n = 480 participants. Even though we initially selected the seeds from our existing network by convenience, according to Salganik and Heckathorn [21], if the chain sampling strategy is appropriately implemented, the estimates remain asymptotically unbiased regardless of the chosen seeds. These individuals, called influencers or seeds, were instructed to invite three other people from their social networks. The survey link was also made available in the research institutions of the coordinators to broaden the diversity of responses.

After the virtual invitation, participants who decided to join the study received a free and informed consent form containing detailed information about the research and a link for further contacts and clarifications.

The responding sample was calibrated based on the population characteristics of the National Household Sample Survey (PNAD) [22]. Variables such as sex, age group, education, and race/skin color were considered for weighting. This statistical procedure is similar to that adopted in other surveys of similar characteristics, such as ConVid [23,24].

### Statistical analysis

To reduce the selection bias of the snowball sampling recruitment procedure, the final sample excluded n = 1,397 respondents from the first two waves (n = 480 initial seeds and their up to three initial invitees). Since information about the contact network was not collected, it was impossible to account for the dependency of observations in the variance estimation.

The final sample of n = 12,136 respondents was then calibrated using data from the National Household Sample Survey (PNAD) of the Brazilian Institute of Geography and Statistics (IBGE) [22] to achieve the same distribution by Brazilian Regions, gender, age group, race/color, and education level as the Brazilian population.

Point prevalences and respective variances of the collected characteristics and variables were estimated, emphasizing the utilization of ICP and stratifying by sociodemographic characteristics. Cross-tabulations between sociodemographic information and the use of ICP were performed, employing variance analysis or Chi-square test to identify differences in utilization of ICP across different socioeconomic and population strata, with a 95% confidence interval.

The odds ratio (OR) for using ICP was estimated using multiple logistic regression models at two hierarchical levels. The first level considered factors unaffected by the pandemic (sociodemographic and economic variables), and the second level incorporated potential effects of the pandemic (work and occupation, health perception, and anxiety). The analyses were conducted using the R statistical package version 4.2.2, considering the sample weight obtained for calibration.

### Ethical considerations

This study is part of the project ‘Use of Integrative and Complementary Practices During the COVID-19 Pandemic–PICCOVID’ approved by the Research Ethics Committees at the *Escola Politécnica de Saúde Joaquim Venâncio/FIOCRUZ/RJ* (34872620.5.0000.5241). The first page of the electronic questionnaire provided information on the study’s purpose, objectives, and duration, along with the participation criteria. Participants were informed that there were no rewards for completing the survey. Additionally, they were assured that their participation was entirely voluntary, that their responses would remain anonymous and be used solely for research purposes, and that the researchers would not be able to identify them. The form began with a question regarding consent to participate; only those who provided consent could proceed to the survey questions.

## Results

The study population consisted of n = 12,136 respondents, predominantly female (53%), under 40 years of age (44.2%), with a predominantly higher education level (59.6%), a household income between R$2,000.00 and R$5,000.00 (U$D 385.00 and U$D 960.00) (34%), and residents of the Central-West region of Brazil (43.7%). Most participants did not experience changes in employment status during the COVID-19 pandemic, continuing their occupational activities either in person or from home, with 22.1% reporting work in essential services (Table 1).

**Table 1:** Sociodemographic, economic, and health characteristics of the study population. Brazil 2020. (n = 12,136).

Variables	%	95% CI
**Sex**		
Male	47.0	41.4–52.6
Female	53.0	47.4–58.6
**Age group (years)**		
18–39	44.2	38.9–49.5
40–59	35.2	30.2–40.6
60 or older	20.6	16.2–25.9
**Race/ethnicity**		
White	49.7	44.3–55.1
Non-White	50.3	44.9–55.7
**Educational level**		
Higher education or more	59.6	54.1–64.8
High school	34.3	29.4–39.7
Up to elementary school	6.1	3.9–9.4
**Region**		
South	7.7	6.5–9.0
Southeast	14.8	13.1–16.6
Central-West	43.7	37.8–49.8
Northeast	26.5	23.4–29.8
North	7.4	6.0–9.1
**Household income**		
More than U$D 1924.00	19.3	13.8–26.2
More than U$D 960.00 and up to U$D 1923.00	16.7	12.9–21.5
U$D 385.00 and U$D 960.00	34.0	28.9–39.4
Up to U$D 385.00	24.4	20.7–28.6
No income	5.6	2.8–11.0
**Health insurance**		
Yes	51.1	45.6–56.5
No/canceled	48.9	43.5–54.4
**Occupation during the pandemic**		
Continued working	30.3	25.9–35.2
Continued working (home office)	26.8	22.2–32.0
Started working	2.7	2.0–3.5
Paid leave or maternity leave	4.3	1.9–9.7
Lost job	8.4	5.9–11.7
It was not working and continued not working	27.4	22.5–33.0
**Essential work activity**		
No	77.9	74.1–81.3
Yes	22.1	18.7–25.9
**Health Perception**		
Excellent/Good	66.5	60.7–71.7
Fair/Poor/Very poor	33.5	28.3–39.3
**Anxiety or nervousness**		
Never/Rarely	41.0	35.9–46.3
Often/Always	59.0	53.7–64.1
**Sleep Quality**		
Not affected/Continued sleeping well	41.1	35.7–46.8
I started having sleep problems	26.8	22.3–31.8
Continued having sleep problems	14.2	11.3–17.7
Had sleep problems, and they worsened	15.2	11.8–19.5
Had sleep problems, and they improved	2.7	1.0–6.8
**Restriction of contact with others**		
No	9.7	7.9–11.7
Yes	90.3	88.3–92.1

Regarding health characteristics, about 66% of the studied population rated their health as excellent or good, and 41.1% reported that the COVID-19 pandemic had not affected their sleep quality. Conversely, approximately 60% reported feeling anxious almost always or always. Just over 51% had health insurance (Table 1).

Nearly all participants (90.3%) reported restricting contact with others during the pandemic (Table 1). Additionally, 51.9% reported having at least one non-communicable chronic disease (NCD) diagnosis, and 42% were vaccinated against H1N1 (data not tabulated).

The overall prevalence of ICP was 61.8% (56.6%– 66.7%), with 24.5% (20.3%– 29.4%) of participants reporting practicing four or more ICPs. The most common ICP were meditation (28.21%, 95% CI: 27.3–29.4), Reiki (21.68%, 95% CI: 21.3–22.7), herbal medicine (28.19%, 95% CI: 27.3–29.9), and aromatherapy (16.48%, 95% CI: 16.1–17.5) (Table 2). In this study, participants were allowed to select as many options regarding ICP types as they desired. However, once they selected an option, the subsequent choices (related to how and why they used each ICP) were restricted to single selections.

**Table 2:** Prevalence and quantity of ICP use (%). Brazil 2020. (n = 12,136).

Variables	%	95% CI
**Quantity of ICP used**		
None	38.2	33.3–43.4
One	16.6	12.7–21.3
Two	9.0	7.4–10.9
Three	11.7	7.5–17.7
Four or more	24.5	20.3–29.4
ICP users	61.8	56.6–66.7
**Types of ICP**		
Meditation	28.2	23.5–33.4
Herbal medicine	28.2	23.7–33.2
Reiki	21.7	17.2–26.9
Aromatherapy	16.5	12.9–20.8
Homeopathy	14.6	10.9–19.2
Flower therapy	14.1	11.0–17.8
Yoga	13.7	10.1–18.4
Apitherapy	11.4	9.5–13.7
Hand imposition	10.6	6.6–16.5
Traditional Chinese Medicine/Acupuncture	7.9	5.4–11.4
Music therapy	7.1	4.6–10.7
Naturopathy	6.1	3.3–11.1
Family constellation	5.6	4.5–7.1
Chromotherapy	5.6	3.3–9.4
Reflexology	5.5	3.1–9.4
Chiropractic	3.7	1.5–9.3
Art therapy	3.6	1.6–8.0
Ayurveda	3.5	2.6–4.8
Bioenergetics	3.1	2.3–4.2
Ozone therapy	2.6	1.0–6.7
Integrative Community Therapy	2.2	1.6–3.1
Hypnotherapy	1.4	0.9–2.2
Circular dance	1.2	0.8–1.7
Anthroposophic medicine	1.2	0.7–2.1
Biodanza	1.0	0.6–1.6
Osteopathy	0.9	0.6–1.3
Geotherapy	0.8	0.4–1.3
Shantala	0.6	0.2–1.5
Social thermalism/Crenotherapy	0.06	0.02–0.1

Among the modalities of ICP, 41.6% practiced alone, 28.4% via the Internet, 27.9% through individual application via videoconference, or in person (23.8%). The main reason for using ICP was to promote health and well-being (82.1%), followed by treating non-communicable diseases (NCDs) (15.1%) and COVID-19 signs and symptoms (10.5%) (data not tabulated).

Considering the adjusted analyses, individuals from the Southeast (AOR = 2,71) and South (AOR = 4.68) regions were significantly more likely to practice some type of ICP than those from the North. Women (AOR = 2.02) and older age groups (AOR3.02) also had a higher likelihood of practicing ICP. No statistically significant associations were observed between ICP and race/ethnicity, educational level, or household income (Table 3).

**Table 3:** ICP use by socioeconomic and demografic variables, frequencies and crude and adjusted Odds Ratios (ORs) in the study population, Brazil 2020 (n = 12,136).

Variables	%	p-value	Crude OR (95% CI)	Adjusted OR (95% CI)
**Sex**				
Male	56.0	0.044	1.00	1.00
Female	66.8		1.77 (1.04–3.01)	2.02 (1.39–2.94)
**Age group (years)**				
18–39	54.8	0.051	1.00	1.00
40–59	64.9		1.63 (0.89–2.98)	1.46 (0.88–2.40)
60 or older	69.1		2.96 (1.45–6.02)	3.02 (1.60–5.71)
**Race/ethnicity**				
White	64.2	0.361	1.00	1.00
Non-White	59.4		1.07 (0.61–1.88)	1.47 (0.90–2.39)
**Educational level**				
Higher education or more	64.5	0.425	1.00	1.00
High school	57.8		0.76 (0.42–1.39)	0.80 (0.50–1.29)
Up to elementary school	56.9		1.11 (0.35–3.51)	0.93 (0.33–2.64)
**Region**				
North	52.3	<0.001	1.00	1.00
Northeast	45.6		0.89 (0.54–1.47)	0.78 (0.43–1.42)
Central-West	71.0		2.24 (0.95–5.27)	2.09 (1.00–4.39)
Southeast	63.4		2.29 (1.40–3.77)	2.71 (1.41–5.18)
South	70.8		3.16 (1.72–5.79)	4.68 (2.13–10.28)
**Household income**				
More than U$D 1923.00	72.8	0.134	1.00	1.00
More than U$D 960.00 and up to U$D 1923.00	64.6		0.68 (0.21–2.20)	0.68 (0.28–1.65)
U$D 385.00 and U$D 960.00	55.2		0.51 (0.18–1.43)	0.72 (0.32–1.65)
Up to U$D 385.00	59.4		0.53 (0.20–1.37)	0.98 (0.43–2.23)
No income	78.7		1.64 (0.36–7.47)	2.14 (0.67–6.84)
**Health insurance**				
No/canceled	56.1	0.012	1.00	1.00
Yes	68.7		1.88 (1.09–3.23)	1.86 (1.11–3.11)

Further adjusted analyses revealed statistically significant higher chances of using ICP among individuals who reported social distancing (AOR = 3.43), those working from home (AOR = 1.89), those who started working during the pandemic (AOR = 2.23), and those working in essential services (AOR = 1.75), compared to reference categories (Table 4). No statistically significant associations were found between the utilization of ICP and health perception, anxiety, and sleep disturbances.

**Table 4:** ICP use by variables that might be affected by COVID-19 in the study population and frequencies and crude and adjusted Odds Ratios (ORs), Brazil 2020 (n = 12,136).

Variables	%	p-value^a^	Crude OR (95% CI)	Adjusted OR (95% CI)
**Occupation during the pandemic**				
Continued working	58.3	0.681	1.00	1.00
Continued working (home office)	66.3		1.54 (0.74–3.21)	1.89 (1.08–3.31)
Started working	58.4		1.66 (0.73–3.75)	2.23 (1.05–4.73)
Took paid leave or maternity leave	70.4		2.39 (0.52–10.94)	1.53 (0.51–4.56)
Lost job	68.9		1.03 (0.58–1.86)	1.46 (0.81–2.64)
It was not working and continued not working	60.9		1.19 (0.60–2.38)	1.31 (0.76–2.26)
**Essential work activity**				
No	60.6	0.382	1.00	1.00
Yes	65.8		1.23 (0.65–2.32)	1.75 (1.05–2.91)
**Health Perception**				
Excellent/Good	64.3	0.354	1.00	1.00
Fair/Poor/Very poor	58.9		0.99 (0.53–1.86)	1.31 (0.82–2.07)
**Anxiety/Nervousness**				
Never/Rarely	66.9	0.111	1.00	1.00
Often/Always	59.3		0.74 (0.45–1.23)	0.58 (0.33–1.01)
**Restriction of contact with others**				
No	42.9	<0.001	1.00	1.00
Yes	66.3		2.76 (1.64–4.64)	3.43 (2.00–5.89)
**Sleep Quality**				
Not affected/Continued sleeping well	62.1	0.254	1.00	1.00
I started having sleep problems	69.8		1.74 (0.95–3.18)	1.97 (1.11–3.49)
Continued having the same sleep problems	55.0		0.66 (0.30–1.44)	0.77 (0.40–1.48)
Had sleep problems, and they worsened	61.5		1.10 (0.50–2.41)	1.28 (0.68–2.41)
Had sleep problems, and they improved	40.8		0.29 (0.05–1.73)	0.30 (0.05–1.69)

^a^ Pearson’s Chi-square test.

## Discussion

Our cross-sectional survey among Brazilian adults showed, that in the first year of the COVID-19 pandemic in Brazil, the prevalence of the use of integrative and complementary practices (ICP) exceeded 60%. A study using data from the 2019 National Health Survey showed that over seven million adults reported using integrative and complementary practices in Brazil, representing a prevalence of 5.4% [17]. The discrepancy in these prevalence rates may be attributed to the different methodologies employed in both studies, as well as the impact of the COVID-19 pandemic on behavior and the search for therapeutic alternatives in a context of social distancing, overwhelmed healthcare services, and the absence of medications and vaccines. Additionally, the present study was more comprehensive regarding the types of ICP compared to the National Health Survey.

In this scenario, there is still a mismatch between the supply and use of ICP within the Unified Health System (UHS) [25]. Although the Ministry of Health of Brazil reports an increase in the availability of integrative and complementary services across the country, the actual utilization of these services through UHS remains relatively low [26]. This indicates that despite the growing presence of ICPs in the public health system, the population has a significant gap in their adoption and use [26].

The demographic profile of the study population associated with a higher prevalence of the use of ICP was female, young, and white, with a high level of education and household income. These associations align with those from studies conducted in other settings [2,27], including a populational study conducted with in-person interviews in Brazil in 2019 [17]. An online survey conducted during the first wave of COVID-19 in the Netherlands showed that ICP was used to prevent and treat COVID-19, most commonly by women and highly educated individuals [4]. Therefore, the study findings highlight inequities in access to practices for low-income populations, considering that part of the ICP offerings in Brazilian public services are still minimal, and this offering may have been discontinued in many cases during the pandemic [25]. Consequently, the low-income and less-educated population was more exposed to the unequal effects of the pandemic [28,29].

A systematic review that included general population surveys and observational studies found significant differences in using ICP between underdeveloped and developed countries during the COVID-19 pandemic [30]. In less developed countries, ICP was often adopted due to limited access to conventional healthcare resources, with interventions seen as essential for boosting immunity and providing holistic care [30]. Conversely, in wealthier nations, the use of ICP was driven more by dissatisfaction with conventional healthcare services and a desire to maintain psychosocial health. For example, Ecuador had the highest proportion of ICP usage, whereas Ethiopia had the lowest [30].

The high prevalence of anxiety (60%) in more than half of the studied population reflects the psychosocial impact of the pandemic. Previous studies have indicated a significant increase in anxiety and stress levels during the COVID-19 pandemic [31–33]. The maintenance of sleep quality for 41.1% of participants can be interpreted as an indicator of resilience or the effectiveness of coping strategies, although nearly a third reported sleep problems, consistent with research documenting sleep disturbances related to pandemic stress [31,34]. Another possibility is that 64.2% maintained employment or started working, corroborating another study that evidenced the interaction between education and occupation as a risk factor for developing insomnia symptoms [28].

Regarding the types of ICP, meditation, Reiki, herbal medicine, and aromatherapy were the most used. These data align with global trends of increasing adoption of integrative health promotion and well-being practices, especially in health crisis contexts [35–38]. The preference for practices such as meditation and Reiki can be attributed to their accessibility, the potential to reduce stress and improve mental health, and the possibility of practicing them in social isolation, particularly relevant during the pandemic [39–42].

The challenges generated during the COVID-19 pandemic demand an increasing integration of various coping and healthcare strategies worldwide and in Brazil. Therefore, the use of herbal medicine, meditation, aromatherapy, and Reiki, among the most prevalent ICPs in the study, can serve as therapeutic support for anxiety and stress situations associated with social isolation, complementing self-care, and enhancing well-being [41,42].

This study utilized non-probabilistic sampling. Post-stratification procedures were used to address the limitations of non-probabilistic sampling and improve representativeness. These procedures adjusted the sample distributions based on demographic variables using data from the National Household Sample Survey to ensure the sample reflected population characteristics [22]. The survey results were compared with estimates from previous and recognized studies, such as the National Health Survey and ConVid [18,19,31]. This comparison showed similarity in most health indicator estimates, suggesting an adequate representation of the Brazilian population in the study sample. Although certain groups with low education levels and limited internet access may have been underrepresented, the survey achieved significant geographic coverage, encompassing all federative units and many municipalities. This indicates a diversity in the sample that can partly offset the recruitment technique’s limitations.

A contextual variable that may have significantly influenced the study results was the heterogeneity in the implementation and access to ICP in Brazil’s regions and social contexts. Several factors, including local health policies, availability of qualified professionals, cultural perceptions, and financial resources, influence the use of ICP. This landscape contributes to notable variations in how ICPs are accessed and practiced by the population. For instance, some regions may have broader integration of ICP in the public health system, while others may face substantial access limitations. Additionally, individuals’ financial capacity to seek ICP in private services may differ, affecting the prevalence and types of practices adopted.

The high adherence to ICP highlights the urgent need for the Brazilian Unified Health System to operate more efficiently and to expand the availability of these practices, particularly during emergencies and epidemiological crises. This is especially important considering their potential to address mental health issues and non-communicable diseases (NCDs) [43]. The prevalent use of ICPs in specific demographic groups underscores the necessity for health policies that recognize and cater to different populations preferences and needs. It is imperative to reduce inequities and broaden the therapeutic range with more culturally accessible offerings, mainly when promoting options for highly vulnerable social groups, providing correct guidance and standards for its use. Also, conducting further research to assess the efficacy and safety of ICP during public health crises, such as COVID-19 is crucial. Developing tailored public health policies for each regions context is not just essential, but also a powerful tool in addressing this issue effectively, providing insights the development of public policies that guide their appropriate use, potentialities, and limitations and inspire further studies.
